# Dried Plasma Spot Based LC–MS/MS Method for Monitoring of Meropenem in the Blood of Treated Patients

**DOI:** 10.3390/molecules27061991

**Published:** 2022-03-19

**Authors:** Haiwei Cao, Yi Jiang, Shaomin Wang, Haihuan Cao, Yanyan Li, Jing Huang

**Affiliations:** 1 Department of Medicine Laboratory, The First Hospital of Jilin University, Jilin University, Changchun 130061, China; caohwjdyy@jlu.edu.cn (H.C.); smwang@jlu.edu.cn (S.W.); 2 Department of Breast Disease, The Second Hospital of Jilin University, Jilin University, Changchun 130061, China; jyi@jlu.edu.cn; 3 Drug and Agricultural Products Laboratory, Changchun Customs Technology Center, Department of Food, Changchun Customs, Changchun 130062, China; haihuan1203@126.com

**Keywords:** meropenem, dried plasma spot, therapeutic drug monitoring, LC–MS/MS

## Abstract

Meropenem (MER) is widely used to treat complicated and serious infections. Therapeutic drug monitoring (TDM) provides a valid clinical tool to avoid suboptimal concentrations and dose–related adverse reactions. However, TDM seems to face challenges since the limited stability of MER in plasma makes transport difficult between clinics and laboratories. Dried plasma spot (DPS) sampling is an attractive but underutilized method for TDM that has the desired features of easy collection, storage, and transport, and overcomes known hematocrit (HCT) issues in dried blood spot (DBS) analysis. This study was designed to investigate a DPS–based liquid chromatography–tandem mass spectrometry (LC–MS/MS) method for quantification of MER. The method was developed and validated for DPS and wet plasma samples. Calibration curves were linear (R^2^ > 0.995) over the concentration range of 0.5–50 µg/mL. Overall accuracy and precision did not exceed 15% and no significant matrix effect was observed. MER has been more stable in DPS than in wet plasma samples. A comparison of DPS and wet plasma concentrations was assessed in 32 patients treated with MER. The results showed that there was no significant difference between the two methods. So the DPS method developed in this study is appropriate and practical for the monitor of MER in the daily clinical laboratory practice.

## 1. Introduction

**Figure 1:**
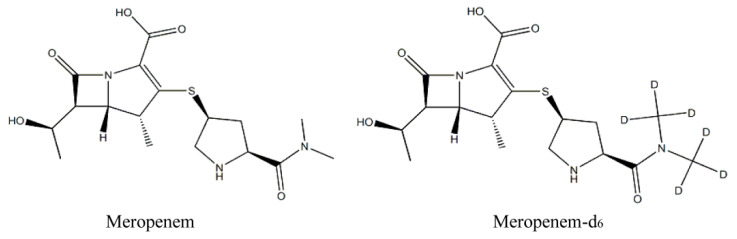
Structures of meropenem and meropenem-d_6_.

Meropenem (MER, Figure 1), a broad-spectrum β-lactam carbapenem antibiotic, has strong antibacterial activity against Gram-negative, Gram-positive, aerobic, facultative, and anaerobic bacteria [1,2]. MER is widely used for empirical treatment of hospitalized patients with bacterial infections, especially in intensive care units [3]. It has been reported that the appropriate administration regimen (i.e., resulting in effective exposure) can improve the clinical success rate [4,5,6]. However, the highly variable and unpredictable pharmacokinetics (PK) of MER in critically ill patients increases a challenge to clinicians in development an optimal antibiotic dosing regimen [7,8]. Sepsis or septic patients may develop the condition of the capillary leak syndrome or augmented renal clearance, which can result in altered PK of a drug [9,10]. The antimicrobial activity of MER is mainly time-dependent and requires that plasma antibiotic concentrations exceed the minimum inhibitory concentration of pathogenic bacteria to achieve sufficient sterilization effect [11]. Therefore, considering the highly variable PK of MER as well as the strong correlation between its exposure and mortality in critically ill patients with infection [7,12,13], therapeutic drug monitoring (TDM) is crucial for optimizing MER concentrations in each patient and improving clinical outcomes [14,15].

Typically, either plasma or serum samples are employed in the TDM of MER and there are no clinically relevant differences between them [16]. Liquid chromatography–tandem mass spectrometry (LC–MS/MS) analysis has been proposed for the quantification of MER in biological matrices due to its better selectivity and sensitivity [17,18,19]. However, the stability of MER is a known issue in serum or plasma samples at room temperature (RT), which increases the challenging for transport and storage of samples and routine therapeutic monitoring of the drug [20,21,22]. Recently, dried blood spot (DBS) or dried plasma spot (DPS) sampling is an alternative sampling strategy in the TDM area that consists of collecting blood samples on filter cards [23,24,25,26]. The practical advantage of using DBS and DPS is that they do not require venipuncture and centrifugation and they can be stored and transported at room temperature (given the stability of analytes), thus reducing the risk of the samples’ degradation [23,27,28].

Of these two microsampling strategies, DPS has been preferred over DBS as the former is free of the hematocrit (HCT) effect. The HCT values may influence sample homogeneity and spot size, dry time, as well as quantitative analysis [29]. However, due to the introduction of a new commercial DPS card as a sampling device, plasma can be separated from blood automatically, avoiding the influence of various inherent HCT levels on quantitative analysis and the problem of uneven blood distribution on the DBS card [30,31,32,33]. In addition, it takes 30 min for plasma samples to dry on a DPS card, while it takes 2–3 h for blood samples to dry on a DBS card.

In this work, we aimed to develop a new method for the determination of MER in DPS. We had compared concentration results obtained in DPS with those obtained in plasma from the same patients to evaluate whether DPS can be a useful matrix for TDM. The current report provides detail on stability of MER in DPS versus plasma under different storage conditions. We have shown, for the first time, a method for the quantification of MER developed on DPS validated for clinical use. The performance of the method allows for rapid and specific measurement of MER in a wide concentration range starting from low (40 µL) volume of plasma samples, with high accuracy and precision. This work is also a proof of concept of the study of DPS sampling for TDM of compounds in biological samples.

## 2. Results and Discussion

### 2.1. Optimization of MS Conditions

MER and meropenem-d_6_ (MER-d_6_) are nitrogen-containing chemicals and they have better response in positive ionization mode. All molecules formed proton adduct ions at *m*/*z* M + 1. Fragment ions were selected in collision-induced dissociation mode at different collision energies from 5 to 80 eV. The MRM acquisitions used the transitions at *m*/*z* 384.0→141.0 for the quantitation of MER. The transition at m/z 390.0→147.0 was used for the quantification of MER-d_6_. The optimal parameters for each compound are given in Section 3.4.2.

### 2.2. Optimization of LC Conditions

Chromatography was performed using a C_18_ column (4.6 × 50 mm, 2.7 µm) since it can offer good retention and excellent peak shape of MER and MER-d_6_. The elimination of the matrix effects and the reduction of the carryover effects could be achieved by using acetonitrile (ACN)–0.1% formic acid in water as the mobile phase with a gradient elution method. Under the optimized LC conditions, the retention time of MER and MER-d_6_ was 3.60 min.

### 2.3. Optimization of Sample Preparation

Protein precipitation with organic solvent was selected for sample processing since it is simple and rapid. For plasma samples, a 30 μL aliquot of human plasma samples, 30 μL internal standard (IS) working solution, 30 μL of diluents and 840 μL ACN were added to precipitate protein. For the DPS samples, a disk was extracted with 30 μL of IS working solution, 30 μL of diluents and 30 µL of ACN. Protein precipitation with 30 times dilution was found to give symmetric peaks with enough sensitivity and ignored matrix effects for MER and MER-d_6_. Therefore, protein precipitation with ACN is an ideal sample preparation assay for this study.

### 2.4. Method Validation

**Figure 2:**
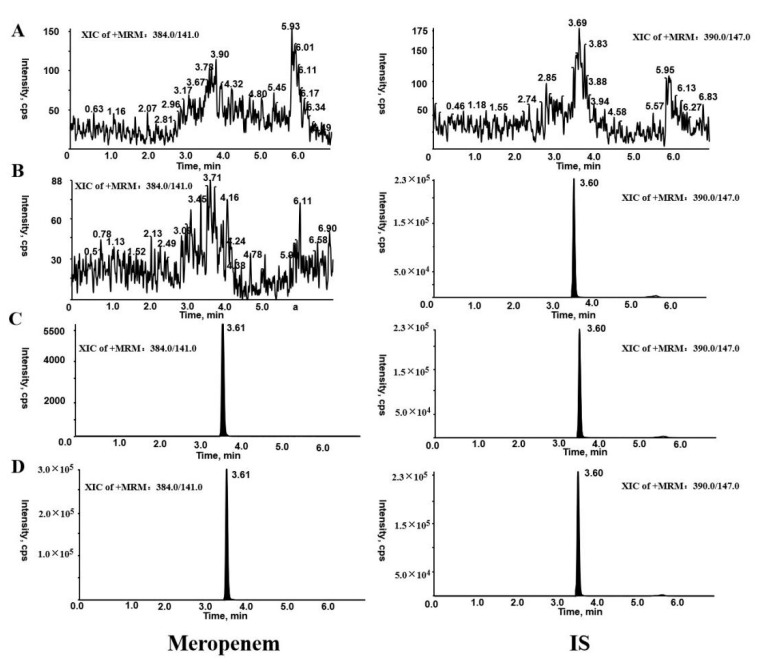
Representative chromatograms of MER and IS: (**A**) blank DPS sample, (**B**) blank DPS sample spiked with IS (1 µg/mL), (**C**) blank DPS sample spiked with MER and IS at the LLOQ (0.5 µg/mL) and IS (1 µg/mL), and (**D**) DPS sample obtained from a patient at 4 h after intravenous administration of MER (23.9 µg/mL).

There were no interfering peaks at their respective retention times for MER and IS in DPS samples (Figure 2). The assay was linear for MER in the range 0.5–50 µg/mL with the regression coefficients of R^2^ ≥ 0.995. A weighting factor of 1/x^2^ provided a better fit for lower-range calibrators. For this study, an lower limit of quantitation (LLOQ) of 0.5 µg/mL was sufficient.

The accuracy and precision of MER determination in DPS and wet plasma samples were investigated using quality control (QC) samples at all concentrations are listed in Table 1. The results of the intra- and inter-day accuracy of DPS and wet plasma samples were 91.5–103.9% and 96.1–103.9%, respectively. The intra- and inter-day precision at QC concentration levels were both <9% for the two methods.

The extraction recoveries and matrix effects for MER in human plasma are shown in Table 2. The data show that the recoveries were in the range of 96.0–104.4% and 98.6–101.4%, respectively in DPS and wet plasma samples. The coefficient of variation (CV, %) of the recovery were <8.2% for both methods. The recovery values were within acceptance limits, and hence, the extraction procedure for MER was reproducible. The matrix effects of MER in DPS and wet plasma samples were 105.2–111.2% and 90.8–100.8%, respectively. No significant ion suppression or enhancement signal was observed in either matrix analyzed.

The stability data of MER under different storage conditions are summarized in Table 3. When stored at room temperature, MER in DPS samples was stable for one day without decline in assay accuracy, with an accuracy range from 91.6 to 92.0%. Wet plasma samples were unstable during the first day with an average degradation of 26%. After seven days at RT, MER showed a significant decay in wet plasma with an average degradation of 72%. However, DPS samples were observed slow decay after seven days under this condition (an average degradation of 31%). Similarly, DPS and wet plasma samples were stored in an incubator at 40 °C for seven days to validate extreme temperatures. The observed fast decay under this condition in both DPS and wet plasma samples may be caused by the high temperature which may have accelerated the decomposition of MER. However, the stability of MER in DPS was significantly better than that in wet plasma. On the other hand, cooled to 4 °C or −20 °C, MER was stable in DPS and wet plasma without statistically significant decays observable over one week or three weeks, respectively. 

The time ranges and temperatures evaluated here were considered relevant for sample transport between clinic and laboratory. As expected, the stability of MER in DPS indeed improved in comparison to wet plasma. The long-term stability of plasma samples containing MER was evaluated by Davide Ferrar [18] and Ronilda D’Cunha [21]. They reported that MER was stable in plasma at −80 °C for up to three months. Our group also repored the long-term stability of MER in a previous study [19]. From this study results, it can be assumed that similar long-term storage stability is realistic on DPS.

The accuracy values of dilution integrity were 96.3–104.6% and 92.5–99.1% for DPS and wet plasma samples, respectively. The precision of the diluted samples was within ±6.9%.

### 2.5. Clinical Application and Method Comparison

**Figure 3:**
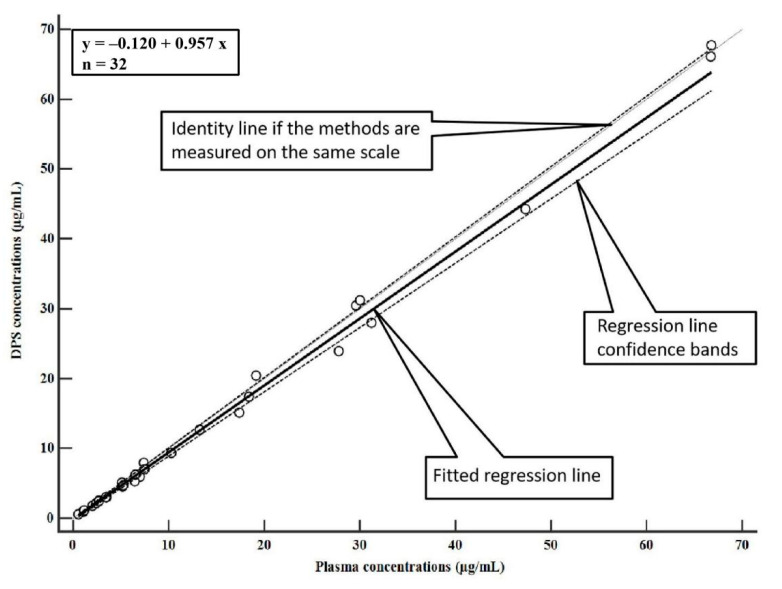
Assessment of assay comparability. Passing–Bablok correlation plots of the concentration of MER from clinical patients. The *X*-axis plots the plasma concentrations (µg/mL) as measured in liquid plasma and the *Y*-axis plots the plasma concentration (µg/mL) as estimated by the DPS method.

**Figure 4:**
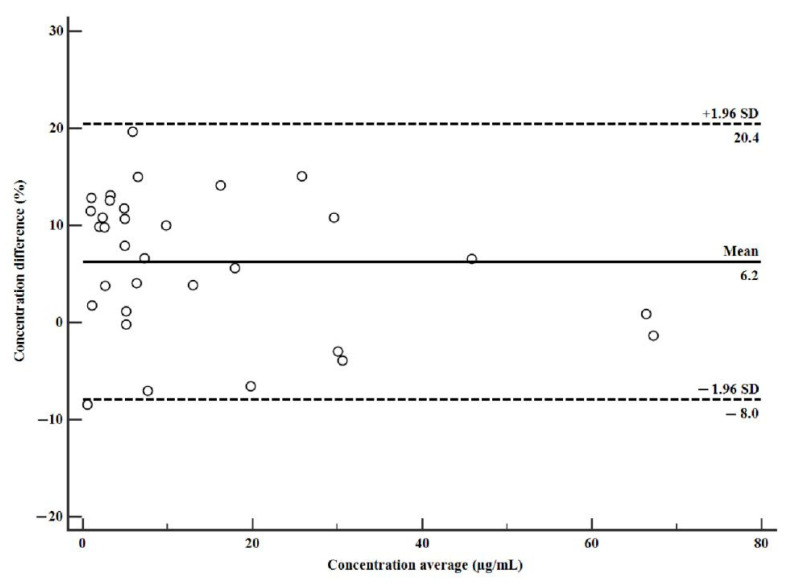
Bland–Altman plots for MER illustrate differences between wet plasma and DPS methods. *X*-axis: average of wet plasma and DPS concentrations; *Y*-axis: difference between the wet plasma and DPS method concentrations expressed as percentage (%). Points between the dashed lines indicate sample pairs that are within the ±1.96SD acceptance range.

The developed DPS assay was applied to drug monitoring of MER in human plasma collected from patients with therapeutic regimens. Representative chromatograms for patient plasma sample are shown in Figure 2. The concentration of MER in 32 human plasma samples analyzed by two methods are illustrated in Table S1. A comparison of the wet plasma and DPS concentrations is shown in Figure 3 and Figure 4. Passing–Bablok plot indicated a strong agreement, no proportional bias, and no constant bias (Figure 3). The corresponding regression equation for MER was y= −0.120 (95% CI, –0.358 to 0.0423) + 0.957x (95% CI, 0.921 to 1.006). We also analyzed the difference between the DPS and wet plasma concentrations using a Bland–Altman plot (Figure 4). The difference between DPS and wet plasma method was 6.2% (95% LoA, −8.0% to 20.4%). Only 1/32 (3.12%) of values fell outside the limits of the agreement; 96.88% of sample pairs had a maximal concentration deviation of ±1.96SD. The concentrations of MER measured from DPS showed no significant biases with those measured from plasma.

## 3. Materials and Methods

### 3.1. Chemicals and Reagents

MER trihydrate (purity 97%) was purchased from Sigma-Aldrich Co. (St. Louis, MO, USA). MER-d_6_ was acquired from Shanghai Pufen Biotechnology Co., Ltd. (Shanghai, China). Formic acid, ACN and methanol (each HPLC grade) were bought from Fisher Scientific (Fairlawn, NJ, USA). Distilled water was bought from Watsons (Changchun, China). Noviplex^®^ plasma prep cards were supplied by Beijing Bio Biotech (Manufactured by Novilytic, West Lafayette, IN, USA).

### 3.2. Calibrators and QC Samples

The stock solution of MER was prepared at concentration of 1 mg/mL with ACN:water (50:50, *v*/*v*) and stored at –20 °C until use. The wet plasma calibration standards (CSs) and wet plasma QC samples were prepared by spiking stock dilutions of appropriate concentration into blank plasma. The prepared CSs were 0.5, 1, 2, 5, 10, 20, 35 and 50 µg/mL of MER. The final concentrations of the QC samples were 1.5, 8 and 40 µg/mL as low-level QC, mid-level QC, and high-level QC samples. A stock solutions of MER-d_6_ as an IS was prepared at a concentration of 1 mg/mL with ACN:water (50:50, *v*/*v*) and diluted with the same solvent to obtain an IS working solutions (10 and 1 µg/mL for wet plasma and DPS analysis, respectively).

CSs and QC samples of the DPS samples were prepared using spiked blank blood aliquots with the same concentration levels. A 40 µL aliquot of spiked blank blood was transferred onto a Noviplex^®^ plasma prep card. After 3 min, the top layer of the DPS card was peeled off and the bottom layer was dried at room temperature for approximately 15 min. The DPS samples were then stored in sealed plastic bags containing desiccant.

### 3.3. Patient Samples

Venous blood samples (containing K_2_EDTA anticoagulate) for patients receiving MER treatment were collected by venipuncture from the First Hospital of Jilin University. After obtaining the blood samples, they were divided into three aliquots, one for the preparation of DPS, another for the HCT measurement, and the last for the wet plasma sample preparation. The aliquots for DPS preparation were pipetted onto Noviplex^®^ plasma prep cards to generate DPS spots. HCT was analyzed for each sample on a Sysmex XN-9000 (Sysmex, Kobe, Japan). The remaining blood sample was centrifuged at 5000 rpm for 3 min to separate plasma which was used for MER quantification in plasma.

### 3.4. Sample Processing and LC–MS/MS Analysis

#### 3.4.1. Sample Processing

For the plasma samples, a plasma aliquot (30 μL) of CSs, QCs and clinical samples was mixed with 30 μL of IS (MER-d_6_ at 10 µg/mL) and 30 μL of diluents (ACN:water, 50:50, *v*/*v*) into a 1.5 mL Eppendorf tube. Extraction of MER from plasma took place using protein precipitation with 810 µL of ACN. The samples were vortex–mixed for 3 min and centrifuged at 13,000 rpm for 5 min. Finally, a 2.0 μL aliquot of the supernatant was injected into the LC–MS/MS system for sample analysis.

For the DPS samples, a disk was removed from the bottom layer of the DPS card, and extracted with 30 µL of IS (MER-d_6_ at 1 µg/mL), 30 µL of diluents and 30 µL of ACN. Samples were vortex–mixed for 3 min and centrifuged at 13,000 rpm for 5 min. A 2.0 μL aliquot of the supernatant was analyzed by LC–MS/MS.

#### 3.4.2. LC–MS/MS Analysis

LC–MS/MS was carried out on a LC–20AD series HPLC system (Shimadzu Corporation, Kyoto, Japan) coupled to a Q-TRAP 5500 mass spectrometer (AB Sciex, Foster City, CA, USA). Briefly, HPLC separation was performed on Agilent Poroshell 120 SB–C_18_ (4.6 mm × 50 mm, 2.7 μm) at 40 °C with a gradient program using eluent A (0.1% formic acid in water) and eluent B (ACN). The gradient program was as follows: 0.0–1.0 min: 4% B; 1.0–2.5 min: 4–95% B; 2.5–4.5 min: 95% B; 4.5–4.6 min: 95–4% B; 4.6–7.0 min: 4% B. The flow rate of mobile phase was set at 0.65 mL/min and the injection volume was 2.0 µL.

An electrospray ionization (ESI) source interface operating in positive mode was used for multiple reaction monitoring (MRM) mode. The operation conditions are following parameters: ion spray voltage, 5500 V; source temperature, 450 °C; curtain gas, 20 psi; ion source gas 1, 60 psi and ion source gas 2, 55 psi; collision gas pressure, medium; entrance potential, 10 V; cell exit potential, 12 V; declustering potential, 80 V; collision energy 18 eV. The monitored MRM transitions were *m*/*z* 384.0→141.0 for MER and *m*/*z* 390.0→147.0 for MER-d_6_.

### 3.5. Method Validation

The method performance was validated based on the Food and Drug Administration (FDA) guidance for bioanalytical method validation [34]. The parameters evaluated were selectivity, linearity, LLOQ, extraction recovery, matrix effect, accuracy, precision, stability, and dilution integrity. The details are described in the Supporting Information.

### 3.6. Method Application

The validated DPS method was applied for the quantification of the MER in 32 plasma samples from patients treated at the intensive care units. This study (Ref No: 2021-736) was approved by the responsible Ethics Committee of the First Hospital of Jilin University. To demonstrate the applicability of the DPS method, the concentrations of MER in the patients measured using the DPS method were compared to those measured using the conventional wet plasma method.

### 3.7. Statistical Analysis

Data processing and graphic presentation were carried out using Microsoft Excel 2016 (Microsoft, Bellevue, Seattle, WA, USA) and MedCalc Version 19.0.7 (MedCalc Software, Mariakerke, Belgium). Data are presented as mean±standard deviation (SD), unless otherwise stated. Agreement between plasma concentration and DPS concentration was determined using the Passing–Bablok regression and Bland–Altman plot analyses. According to European Medicines Agency guideline [35], at least 67% of the sample pairs (DPS concentrations, wet plasma concentrations) should deviate within 1.96SD of the mean difference to use an alternative method in exchange reliably.

## 4. Conclusions

In this paper, we developed and validated a DPS based LC–MS/MS method for the quantification of MER in human blood samples. The method was validated in accordance with the FDA guidelines and it was applied to clinical samples for the accurate quantification of MER. Compared to plasma, MER is more stable in DPS that may simplify sample transport from remote sites to tertiary care center where access to LC–MS/MS instrumentation is more likely. Many small clinical facilities, especially those located in remote areas, usually do not have sufficient facilities to quantify the drug levels required internally or to store and transport plasma samples to analytical laboratories at deep freezing temperatures [26]. In this circumstances, DPS sampling is the best possible choice. The results of our study on the stability of MER in DPS under different storage conditions provide the important information about the required sample storage conditions to the clinicians and laboratories. This work is a proof of concept for using DPS method for quantitative analysis of compounds in biological samples.

## Figures and Tables

**Table 1:** Precision and accuracy of DPS and wet plasma samples.

Species	Spiked Concentration (µg/mL)	Intra-Day (*n* = 6)		Inter-Day (*n* = 18)	
		Accuracy (%)	Precision (CV, %)	Accuracy (%)	Precision (CV, %)
DPS	0.5	97.0 ± 6.5	6.7	96.8 ± 7.1	7.4
	1.5	91.5 ± 6.0	6.5	92.0 ± 5.6	6.1
	8	103.9 ± 8.9	8.6	104.3 ± 7.4	7.1
	40	99.4 ± 3.6	3.7	100.8 ± 3.5	3.5
Wet plasma	0.5	96.1 ± 6.6	6.9	100.2 ± 7.4	7.1
	1.5	97.2 ± 5.2	5.3	97.0 ± 4.5	4.6
	8	99.8 ± 7.2	7.3	103.9 ± 7.3	7.0
	40	101.9 ± 3.1	3.0	97.9 ± 6.1	6.2

**Table 2:** Recovery and matrix effect of MER from DPS and wet plasma samples (*n* = 6).

Species	Spiked Concentration (µg/mL)	Recovery (%)		Matrix Effect (%)	
		Average	CV	Average	CV
DPS	1.5	104.4 ± 6.6	6.3	105.2 ± 6.6	6.2
	8	97.6 ± 5.3	5.5	108.4 ± 3.8	3.4
	40	96.0 ± 5.5	5.7	111.2 ± 5.4	3.7
Wet plasma	1.5	101.4 ± 7.7	7.6	91.9 ± 3.7	4.1
	8	99.1 ± 4.5	4.5	100.8 ± 8.6	8.5
	40	98.6 ± 8.0	8.2	90.8 ± 6.8	7.4

**Table 3:** Stability of MER in DPS and wet plasma under various storage conditions (percentage of nominal concentration, *n* = 3).

Condition	DPS (Nominal Concentration, µg/mL)		Wet Plasma (Nominal Concentration, µg/mL)	
	1.5	40	1.5	40
R.T for 1 d	91.6 ± 1.4	92.0 ± 4.9	71.6 ± 6.5	76.4 ± 4.5
R.T for 2 d	72.2 ± 5.2	74.3 ± 4.8	60.3 ± 2.0	64.0 ± 0.9
R.T for 3 d	76.1 ± 6.9	74.3 ± 4.0	49.4 ± 1.4	54.5 ± 2.8
R.T for 4 d	72.0 ± 3.0	73.5 ± 7.2	39.3 ± 1.8	43.1 ± 1.5
R.T for 7 d	70.3 ± 5.2	68.3 ± 5.1	27.5 ± 3.8	28.1 ± 0.9
40 °C for 1 d	64.3 ± 1.5	74.7 ± 2.6	60.4 ± 2.0	55.8 ± 1.4
40 °C for 2 d	43.3 ± 3.4	60.8 ± 2.5	11.6 ± 0.5	14.8 ± 0.2
40 °C for 3 d	37.5 ± 0.8	52.9 ± 0.1	6.6 ± 0.3	3.7 ± 0.1
40 °C for 7 d	31.7 ± 3.3	40.9 ± 2.0	4.0 ± 0.4	0.1 ± 0.0
4 °C for 1 w	100.4 ± 2.1	103.8 ± 10.0	87.0 ± 3.1	93.6 ± 6.5
20 °C for 3 w	93.2 ± 6.0	101.5 ± 9.2	106.7 ± 3.1	98.4 ± 2.8

R.T, room temperature(25 °C).

## Data Availability

The data presented in this study are available on request from the corresponding author.

## Supplementary Materials

The following supporting information can be downloaded at www.mdpi.com/article/10.3390/molecules27061991/s1, Method Validation and Table S1: Concentrations of MER in 32 human plasma samples analyzed by DPS and wet plasma methods.
