# Supplementary material for: Dried Plasma Spot Based LC–MS/MS Method for Monitoring of Meropenem in the Blood of Treated Patients
Source: Molecules. 2022 Mar 19;27(6):1991. doi: 10.3390/molecules27061991 (PMC8949976; doi:10.3390/molecules27061991)
Supplement: Supplementary file 1 [file molecules-27-01991-s001.zip › molecules-1617425-supplementary.pdf]

molecules

MDPI

Supplementary material

Dried Plasma Spot Based LC–MS/MS Method for Monitoring of Meropenem in the Blood of Treated Patients

Haiwei Cao [1,†], Yi Jiang [2,†], Shaomin Wang [1], Haihuan Cao [3], Yanyan Li [1,*] and Jing Huang [1,*]

[1] Department of Medicine Laboratory, The First Hospital of Jilin University, Jilin University, Changchun 130061, China; caohwjdyy@jlu.edu.cn (H.C.); smwang@jlu.edu.cn (S.W.)
[2] Department of Breast Disease, The Second Hospital of Jilin University, Jilin University, Changchun 130061, China; jyi@jlu.edu.cn
[3] Drug and Agricultural Products Laboratory, Changchun Customs Technology Center, Department of Food, Changchun Customs, Changchun 130062, China; haihuan1203@126.com
* Correspondence: yanyanli@jlu.edu.cn (Y.L.); huangj@jlu.edu.cn (J.H.)
† These authors contributed equally to this work.

3.5 Method Validation

3.5.1 Selectivity, linearity and LLOQ

Selectivity was evaluated by analyzing the plasma and blood samples from six healthy volunteers. Selectivity was also investigated by spiking blank samples with IS or a standard at the LLOQ to check the signal intensity of the analyte. An interference of <20% of the LLOQ level (<5% for the IS) at the observed retention time window of the analyte (30 s) was acceptable for selectivity.

Linearity was evaluated by analyzing the calibration standards in both DPS and plasma samples (ranging 0.5–50 μg/mL) on three non-consecutive days. Calibration curves were constructed by plotting peak area ratio (y) of MER/IS versus its nominal concentration (x) using a $1/x^2$ weighting factor. An acceptable determination coefficient ($R^2 \geq 0.995$) was obtained. The LLOQ was defined as the lowest concentration of the calibration curve. The reproducibility at the LLOQ level was evaluated by consecutively injecting six processed samples at the LLOQ level and comparing its precision and accuracy against the established linearity. A maximum variation of ≤20% was observed.

3.5.2 Precision and accuracy

Precision (coefficient of variation, CV) and accuracy (relative error) were estimated by analyzing six sets of replicates of QC samples of DPS and plasma. Intra- and inter-day precision and accuracy were assessed by analyzing QC samples at four different concentration levels (0.5, 1.5, 8, and 40 μg/mL) on three separate days. The precision was acceptable if it did not exceed 15% (20% for LLOQ) of the CV, and the accuracy was acceptable if it was within 85-115% (80–120% for LLOQ) of the nominal concentration.

3.5.3 Matrix effect and recovery

The extraction recovery and matrix effect of the method were assessed by analyzing six samples at three different levels (1.5, 8 and 40 μg/mL). For this validation, three separate sets were prepared. The first set (Set A) was prepared by adding QC and IS (1 μg/mL) samples into blank DPS samples and drying prior to extraction. The second set (Set B) was prepared by spiking the QC working solutions and IS after the DPS extraction process; The third set (Set C) was acquired by adding QC working solutions and IS into an equivalent volume of pure solutions. The extraction recovery and matrix effect can be evaluated by the following formula: recovery (%) = A/B × 100; and matrix effect

(%) = B/C × 100. The extraction recovery and matrix effects of MER from wet plasma were evaluated similarly.

3.5.4 Stability and dilution integrity

The stability of MER in DPS and wet plasma under various storage conditions were evaluated. QC samples with 1.5 and 8 μg/mL MER were tested on days 1, 2, 3, 4, and 7 of storage at room temperatureat (25 °C). Stability in extreme conditions, 40 ℃ for 1, 2, 3, 7 days, 4 ℃ for 1 week, and –20 ℃ freezer for 3 weeks were also tested. The DPS card was stored in a sealed bag with a desiccant, and the plasma was stored in a sealed Eppendorf tube. MER was considered stable if the mean accuracy values were within ±15.0% and the CV was within 15.0%.

Dilution integrity was validated by spiking the matrix with an MER concentration greater than the upper limit of quantitation and diluting this sample with a blank matrix. An MER solution equal to 80 μg/mL was diluted 5–fold with a blank matrix to obtain a final concentration (16 μg/mL) that fell within the calibration interval. Dilution integrity was demonstrated using six replicates.

Table S1 Concentrations of MER in 32 human plasma samples analyzed by DPS and wet plasma methods

Sample ID	DPS (μg/mL)	Wet plasma (μg/mL)
sample1	0.932	1.06
sample2	0.909	1.02
sample3	1.83	2.02
sample4	3.06	3.49
sample5	2.19	2.44
sample6	4.57	5.14
sample7	5.99	6.96
sample8	9.32	10.3
sample9	12.7	13.2
sample10	20.4	19.1
sample11	17.4	18.4
sample12	28	31.2
sample13	23.9	27.8
sample14	0.594	0.546
sample15	2.99	3.39
sample16	1.11	1.13
sample17	15.1	17.4
sample18	44.3	47.3
sample19	67.7	66.8
sample20	66.1	66.7
sample21	30.5	29.6
sample22	31.2	30
sample23	5.06	5.12

sample24	4.74	5.13
sample25	5.31	6.47
sample26	6.24	6.5
sample27	2.58	2.68
sample28	2.43	2.68
sample29	5.12	5.11
sample30	4.71	5.24
sample31	7.93	7.39
sample32	6.99	7.47
